# Transcriptional profiling of long non-coding RNAs and novel transcribed regions across a diverse panel of archived human cancers

**DOI:** 10.1186/gb-2012-13-8-r75

**Published:** 2012-08-28

**Authors:** Alayne L Brunner, Andrew H Beck, Badreddin Edris, Robert T Sweeney, Shirley X Zhu, Rui Li, Kelli Montgomery, Sushama Varma, Thea Gilks, Xiangqian Guo, Joseph W Foley, Daniela M Witten, Craig P Giacomini, Ryan A Flynn, Jonathan R Pollack, Robert Tibshirani, Howard Y Chang, Matt van de Rijn, Robert B West

**Affiliations:** 1 Department of Pathology, Stanford University School of Medicine, 269 Campus Drive, Stanford, CA 94305-5324, USA; 2 Department of Pathology, Beth Israel Deaconess Medical Center, Harvard Medical School, 330 Brookline Avenue, Boston, MA 02215, USA; 3 Department of Genetics, Stanford University School of Medicine, 269 Campus Drive, Stanford, CA 94305-5120, USA; 4 Department of Biostatistics, University of Washington, 1705 NE Pacific Street, Seattle, WA 98195-7232, USA; 5 Program in Cancer Biology, Stanford University School of Medicine, 269 Campus Drive, Stanford, CA 94305-5456, USA; 6 Howard Hughes Medical Institute and Program in Epithelial Biology, Stanford University School of Medicine, 269 Campus Drive, Stanford, CA 94305, USA; 7 Department of Statistics, Stanford University, 390 Serra Mall, Stanford, CA 94305-4065, USA; 8 Department of Health Research and Policy, Stanford University School of Medicine, 269 Campus Drive, Stanford, CA 94305-5405, USA

**Keywords:** 3SEQ, FFPE, human cancer, intergenic transcripts, lncRNAs, novel transcripts, solid tumors, transcriptional profiling

## Abstract

**Background:**

Molecular characterization of tumors has been critical for identifying important genes in cancer biology and for improving tumor classification and diagnosis. Long non-coding RNAs, as a new, relatively unstudied class of transcripts, provide a rich opportunity to identify both functional drivers and cancer-type-specific biomarkers. However, despite the potential importance of long non-coding RNAs to the cancer field, no comprehensive survey of long non-coding RNA expression across various cancers has been reported.

**Results:**

We performed a sequencing-based transcriptional survey of both known long non-coding RNAs and novel intergenic transcripts across a panel of 64 archival tumor samples comprising 17 diagnostic subtypes of adenocarcinomas, squamous cell carcinomas and sarcomas. We identified hundreds of transcripts from among the known 1,065 long non-coding RNAs surveyed that showed variability in transcript levels between the tumor types and are therefore potential biomarker candidates. We discovered 1,071 novel intergenic transcribed regions and demonstrate that these show similar patterns of variability between tumor types. We found that many of these differentially expressed cancer transcripts are also expressed in normal tissues. One such novel transcript specifically expressed in breast tissue was further evaluated using RNA *in situ *hybridization on a panel of breast tumors. It was shown to correlate with low tumor grade and estrogen receptor expression, thereby representing a potentially important new breast cancer biomarker.

**Conclusions:**

This study provides the first large survey of long non-coding RNA expression within a panel of solid cancers and also identifies a number of novel transcribed regions differentially expressed across distinct cancer types that represent candidate biomarkers for future research.

## Background

The differentially expressed genes from hundreds of cancer profiling studies over the last several years have yielded numerous biomarkers that have improved the subtyping, classification and diagnosis of tumors for both research and the clinic [1-3]. Because of the extent to which cancers have been studied by gene microarrays, protein-coding genes have been well studied in this context. Recent studies looking beyond protein-coding genes have shown that microRNAs (miRNAs) can show higher specificity as biomarkers for some applications than protein-coding genes. As the landscape of long non-coding RNAs (lncRNAs) grows, studies have found that lncRNAs also tend to show more tissue-specific expression than protein-coding genes [4]. This property of lncRNAs makes them highly attractive as tissue-specific biomarkers.

LncRNAs, generally defined as having a size greater than 200 nucleotides, make up a diverse group of non-coding RNAs that are distinct from miRNAs. Until recently, very few lncRNAs were annotated within the human genome. Now, various groups have developed independent catalogs of human lncRNAs [4-11]. These range from the lncRNA database containing a few hundred high-confidence, experimentally validated lncRNAs [12] to the largest of the lncRNA catalogs, compiled by the GENCODE group, who combined manual curation, computational analysis and targeted experimental validation of the GENCODE transcript database to predict more than 14,000 lncRNA transcripts from roughly 9,000 gene loci [5,13]. Despite the thousands of human lncRNAs now predicted, few lncRNAs have been well characterized to date, so little is known about the expression patterns of most lncRNAs in more than a handful of cell types.

Recent analyses suggest that lncRNAs may play key roles in cancer [14,15]. LncRNAs have been shown to play an important role in epigenetic gene regulation and cellular differentiation [16-18], both of which are processes frequently deregulated in cancer. Targeted evaluation of the lncRNA Hox antisense intergenic RNA (HOTAIR) in a set of clinical breast cancer specimens showed that HOTAIR expression was associated with breast cancer metastasis and acts by disrupting polycomb repressive complex 2-dependent gene silencing [19,20]. Despite the growing expectation that numerous lncRNAs are important regulators within both normal and cancer biology, no study to date has performed a survey of lncRNA expression across a broad range of human cancer types. We hypothesized that the usage of lncRNAs may be different between normal tissues and cancer as well as between various cancers. To address this, we chose to profile lncRNA expression across a range of primary human tumors and compare expression patterns against a small sampling of normal tissues. A broad collection of cancers offers a rich resource for studying the usage of known lncRNAs across a range of cancer biologies and also provides the opportunity to identify new lncRNA candidates.

High-throughput sequencing provides a number of advantages over other profiling methods for this type of study. Sequencing of polyA+ RNA from the various cancer samples allows for the profiling of transcriptional patterns of protein-coding mRNAs as well as both known and novel lncRNAs, and any other relatively long polyadenylated transcripts. Traditional RNA sequencing (RNAseq) has limitations when profiling numerous primary tumor samples, including the need for high quality RNA, which is difficult to obtain from formalin-fixed clinical specimens, and the expense associated with deep sequencing of many samples.

Instead, we chose to profile a diverse set of archived human cancers using 3'-end sequencing for expression quantification (3SEQ) [21,22]. This variation on the traditional RNAseq method enriches for 3' ends of transcripts by relying on polyA+ selection of fragmented RNA. Only the 3'-end-most polyadenylated fragment of each transcript is isolated and sequenced, making the technique especially suitable for archived samples with fragmented RNA. This 'sequence tag' approach (not unlike expressed sequence tag (EST) libraries) also significantly reduces the sequencing depth necessary relative to RNAseq, because RNAseq targets the entire length of each transcript. By reducing the sequencing to only a 3' fragment from each transcript using a polyA+ selection, 3SEQ allowed us to substantially reduce the depth of sequencing required to discover and quantify rare RNAs and allowed us to profile many more samples than would have been possible using traditional RNAseq. Additionally, because of its strand-specific libraries, 3SEQ also provided directional information for each sequence read, allowing separate quantification of transcripts on both strands.

Given our interest in exploring lncRNA expression across a broad range of solid tumors and identifying new transcripts associated with various cancer types, we chose to profile a diverse set of solid human tumors representing adenocarcinomas and squamous cell carcinomas from different organs, as well as a variety of soft-tissue sarcomas.

Although all of these cancer subtypes have been previously evaluated by gene microarray, this study represents the first unbiased sequencing-based screen for lncRNAs and other long novel transcripts across this diverse set of cancer subtypes.

## Results

### 3SEQ profiling of lncRNAs and novel transcribed regions

To profile known lncRNAs as well as identify and quantitate novel transcripts across a range of solid cancers, we performed 3SEQ, a 3'-strand-specific sequencing technique for the quantification of polyA+ RNA [21], to obtain global gene expression patterns in 64 solid tumors, representing 17 diagnostic classes of both sarcomas and carcinomas. Over 1.7 billion total short sequence reads were generated, resulting in more than 409 million reads that mapped uniquely to the human genome (Table S1 in Additional file 1). Because the 3SEQ method captures only the 3' polyadenylated ends of RNA fragments and directionally sequences the start of the fragments, the resulting sequencing reads cluster into strand-specific peaks when mapped to the reference human genome. Initial analysis of the uniquely mapping reads from the 66 libraries (two tumor samples had duplicate libraries) demonstrated that most sample libraries possessed reads from at least 24,000 of the 37,576 RefSeq transcripts, and this number did not increase significantly with deeper sequencing (Figure S1 in Additional file 2). Raw and processed gene expression data are available in GEO [GEO:GSE28866].

Because we were particularly interested in the expression patterns of unannotated transcribed regions and known lncRNAs, including recently discovered lncRNAs, we adopted a systematic unbiased analysis approach to identify clusters of sequencing reads across the genome, independent of gene annotations. After pooling reads from all samples, we applied a sliding-window peak-calling algorithm to identify genomic regions enriched for sequencing reads (see Methods). Our initial analysis revealed 54,511 clusters of sequence reads with at least 150 reads across the 66 samples. These peaks, situated across the genome, had a median length of 196 base pairs (bp), and represented same-strand clustered sequence reads. Further filtering and increasing the threshold for peak inclusion resulted in a dataset containing 36,048 peaks with a median enrichment of 74-fold above background in at least one sample (see Methods).

**Figure 1:**
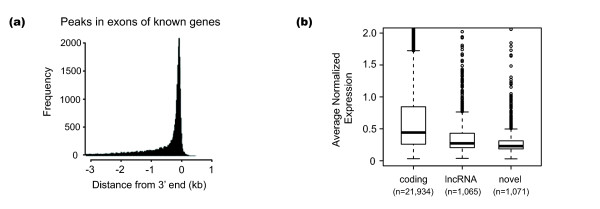
**Distribution and mean expression of 3SEQ peaks**. **(a) **The distribution plot shows a tight cluster of exonic peaks approximately 275 bp upstream of the 3' end of known genes (*n *= 29,024 peaks in known exons; distances are based on genomic coordinates and not the spliced transcriptome). **(b) **Boxplots show the distribution of mean expression levels for each peak by peak category. Raw sequence count data was normalized by dividing each value by the sample mean, and then taking the square root. Boxes range from the first to the third quartiles. Median expression is marked with a line. Mean values are 0.799, 0.407 and 0.324 for coding, lncRNA and novel transcripts, respectively. Plots are truncated to show mean expression values less than 2. Outlier peaks show expression as high as 17.9.

We first compared these peaks with a collection of known protein-coding genes (compiled from RefSeq and University of California, Santa Cruz (UCSC) known genes). The genomic coordinates for 21,934 of the peaks at least partially overlapped exons from annotated protein-coding genes. When the peaks were compared with a broader definition of known transcripts (coding and non-coding from RefSeq, UCSC known genes, GENCODE and UCSC all_mRNA), 23,449 peaks were situated within the 3'-most exon of at least one annotated transcript. When non-3' exons were included in the analysis, 25,258 peaks were determined to be exonic. Because the 3SEQ sequence reads were from RNA fragments ranging between 200 and 300 bp in length, it was not unexpected to observe a tight distribution of exonic peaks approximately 275 bp upstream of the 3'-end of known genes (Figure 1a).

Although theoretically 3SEQ provides sequence reads against all polyA+ transcripts present in the samples and we observed thousands of peaks within protein-coding genes, we focused our analysis on those peaks that overlap known lncRNA genes or are situated in intergenic regions and may correspond to novel transcripts. Four of the larger recent lncRNA catalogs were combined and used as the most current collection of known lncRNA transcripts [4,10,11,13]. After the 21,934 peaks from coding genes were excluded, 1,065 peaks overlapped known lncRNA transcripts. These lncRNAs, most of them identified in normal tissues or cancer cell lines and previously unstudied in tumor samples, were expressed in at least one of the solid tumors in our survey (Tables S2 and S3 in Additional file 1). The vast majority of the remaining peaks could be ascribed to either exons or introns within other known transcripts including non-coding transcripts, or regions within 5 kb downsteam of known transcripts that likely represented alternative 3'-ends for known genes (Table S2 in Additional file 1). Following removal of peaks within or near known transcripts, we defined a list of 1,071 intergenic peaks greater than 5 kb from the nearest known transcript (Tables S2 and S4 in Additional file 1). These intergenic peaks represented novel transcribed regions at what could be the 3'-ends of new independent transcripts. Based on the characteristics of the peaks from known genes, we infer that the intergenic peaks represented sequenced regions located approximately 275 bp upstream of the 3'-end of novel polyA+ RNA transcripts.

Although knowledge of the novel transcripts' start, stop and exon structure is not necessary for analysis of the 3SEQ peak expression data, we performed a preliminary analysis of the transcript structure of the novel intergenic peaks by comparing them with predicted transcripts assembled from a large, publically available RNAseq dataset from 16 normal human tissues. Scripture was run on Illumina's BodyMap RNAseq dataset to predict assembled transcripts from the RNAseq reads [6,23]. Because this reference set was assembled using only a handful of normal samples, it was not surprising that only 402 (37.5%) of the novel intergenic 3SEQ peaks overlapped with novel transcripts from the normal dataset. Interestingly, however, only 72 intergenic peaks overlapped BodyMap multi-exon transcripts with predicted splice sites. Similar to results described by van Bakel and colleagues [11], 330 (82.1%) of the overlapping 3SEQ intergenic peaks corresponded to predicted single-exon transcripts in the BodyMap dataset. Similarly, 558 (52%) of our novel peaks overlapped with ESTs, but only 88 (8%) overlap with spliced ESTs. Single-exon transcripts have not traditionally been classified as lncRNAs, but their polyA+ status separates them from traditional miRNAs.

To further evaluate our intergenic transcripts with respect to other annotations, we compared our novel peaks with a variety of datasets (see Table S4 in Additional file 1 and Methods). Although 269 intergenic peaks (25%) overlapped conserved regions, 588 (55%) contained at least some repetitive sequences. A handful of the intergenic peaks corresponded with known genes from GENCODE that are absent from the RefSeq and UCSC gene annotations: 23 (2.1%) and 13 (1.2%) intergenic peaks overlapped GENCODE transcripts on the sense and antisense strands, respectively. Another 23 (2.1%) are within introns of GenBank mRNAs. Likewise, a small number (25, 2.3%) of the intergenic peaks overlapped ribosomal RNA pseudogenes, while another 31 (2.9%) overlapped other pseudogenes. None of the intergenic peaks overlapped annotated small nucleolar RNAs or miRNAs, but 66 of the peaks (6.2%) were antisense to other 3SEQ peaks. Even though the classification and functional status of most of the 3SEQ intergenic peaks remained unclear, we were interested in determining if any of these transcripts were associated with specific cancer classes and could be used as cancer biomarkers.

### Expression analysis of lncRNAs and novel transcribed regions in solid tumors

After identifying lncRNAs and new transcripts expressed in our panel of tumors, we explored how these RNAs were expressed across the different types of cancer surveyed. The sequencing analysis comprised 66 libraries from 64 independent cancer samples representing 17 different diagnostic classes. Between two and seven samples were selected to represent each diagnosis, with roughly half of the samples representing carcinomas (adenocarcinomas, squamous cell carcinomas and others) and the other half representing more rare sarcomas. Because the 3SEQ peaks were identified based on pooled reads from all of the cancer samples, the peaks could represent RNAs expressed in any number of the samples. To assess sample expression levels, we tallied the total number of 3SEQ reads within each peak for each sample, and the raw read counts were normalized between samples to account for the sequencing depth of each library (see Methods).

**Figure 2:**
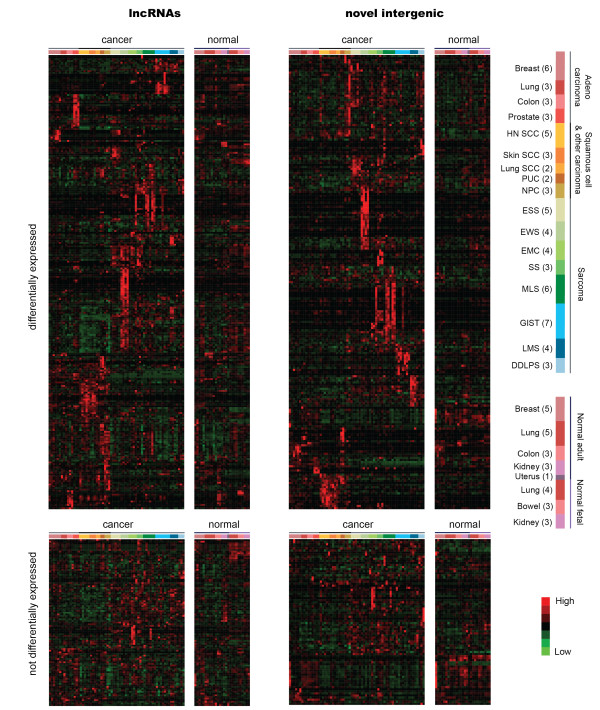
**Variably expressed lncRNAs and novel intergenic transcripts**. Heatmaps illustrating the 368 lncRNAs (left) and 297 novel transcripts (right) with variable expression as defined by standard deviation >0.25 across 66 cancer samples. Transcripts with differential expression in at least one of the 17 two-class SAM analyses (top) were clustered separately from those transcripts not significantly differentially expressed (bottom). Normalized read data were median centered, hierarchically clustered and plotted on a low (green) to high (red) heatmap. Samples are grouped by cancer type; the number in parentheses indicates the number of libraries for each cancer type. Red and pink is used for libraries made from adenocarcinomas of breast, lung, colon and prostate, as well as normal breast, lung and colon. Orange and yellow show squamous cell carcinomas of the head and neck, skin, lung and other carcinomas: papillary urothelial carcinoma and nasopharyngeal carcinoma. Green indicates sarcomas with known translocations: endometrial stromal sarcoma, Ewing's sarcoma, extraskeletal myxoid chondrosarcoma, synovial sarcoma and myxoid liposarcoma. Blue shows other sarcomas: gastrointestinal stromal tumor, leiomyosarcoma and dedifferentiated liposarcoma. Normal samples and cancer samples were combined for hierarchical clustering, but are displayed separately for clarity. Samples are ordered according to Table S1 in Additional file 1. Breast, breast invasive ductal carcinoma; colon, colon adenocarcinoma; DDLPS, dedifferentiated liposarcoma; EMC, extraskeletal myxoid chondrosarcoma; ESS, endometrial stromal sarcoma; EWS, Ewing's sarcoma; GIST, gastrointestinal stromal tumor; HN SCC, head and neck squamous cell carcinoma; LMS, leiomyosarcoma; Lung, lung adenocarcinoma; Lung SCC, lung squamous cell carcinoma; MLS, myxoid liposarcoma; NPC, nasopharyngeal carcinoma; prostate, prostate adenocarcinoma; PUC, papillary urothelial carcinoma; Skin SCC, skin squamous cell carcinoma; SS, synovial sarcoma.

Globally, the average expression levels of the lncRNA peaks and the intergenic peaks were reduced relative to the expression of coding genes (1.96-fold difference for lncRNAs and 2.46-fold difference for the novel transcripts; *P *<2.2 × 10^-16^; Figure 1b). Average lncRNA expression was slightly higher than average expression of the novel transcripts (*P *<0.0005; Figure 1b). To examine those transcripts with higher, more variable expression levels across samples, we selected peaks with a standard deviation greater than 0.25 (roughly the median expression level of lncRNA and novel transcripts) across the cancer samples. This focused our analysis to 368 lncRNA peaks and 297 novel transcribed regions. Strikingly, samples of the same diagnosis tended to show similar expression levels, with various combinations of the cancer types showing expression across the peaks surveyed (Figure 2). Significant expression differences were shown between the carcinomas and sarcomas for 483 of the lncRNAs and 394 of the novel peaks (false discovery rate (FDR) <0.05). Other peaks showed specificity for various combinations of the adenocarcinomas or the squamous cell carcinomas. Many peaks, however, appeared to show even more specificity, with expression in only a small fraction of the cancer types.

To better characterize this specificity and identify which of the above peaks was differentially expressed in at least one diagnostic subtype, we performed a two-class significance analysis using significance analysis of microarrays (SAM) [24] for each of the 17 cancer classes. This allowed us to identify peaks differentially expressed in each diagnostic class versus all other classes. These analyses revealed 267 lncRNAs (72.6%) and 217 novel peaks (73.1%) were differentially expressed in at least one cancer type with an FDR of 0.05 (Figure 2 and Tables S3, S4 and S5 in Additional file 1). Of these peaks showing significant differential expression, most peaks (202 lncRNAs and 189 novel peaks) were significantly expressed in only one of the 17 diagnostic classes, making these peaks cancer-type specific with respect to the other cancers surveyed.

To confirm the class-specific differential expression measured by 3SEQ for a subset of 3SEQ peaks, we performed quantitative RT-PCR (qRT-PCR) on 23 peaks in a subset of the samples profiled by 3SEQ. In this qRT-PCR analysis, 19 of the 23 peaks had elevated expression in samples from the diagnostic subtype that previously had shown elevated expression by 3SEQ (Figure S2 in Additional file 2).

### lncRNA and novel transcript expression in normal tissues

Although we observed many lncRNAs and novel transcripts expressed specifically in some cancer types with respect to others, it was unclear if the transcripts were specific to cancer or instead were normally expressed transcripts, likely possessing a function within normal tissue biology, that were also expressed in cancers. To address how the cancer-expressed lncRNAs and novel transcribed peaks identified above were expressed in normal tissues, we performed 3SEQ on 27 normal samples, including 10 fetal samples from bowel, lung and kidney at 13 to 15 weeks and 17 adult tissues from normal breast, colon, kidney, lung and uterus. The number of 3SEQ reads was determined for each of the previously identified peaks, and the normalized expression data were analyzed. Just as with expression patterns across the cancer subtypes, lncRNA and novel peak expression showed a number of patterns in the normal tissue samples: some did not appear to be expressed or were expressed at very low levels, some were expressed across most normal samples, some were limited to adult samples, some to fetal samples, and some showed more tissue-specific expression patterns (Figure 2).

**Figure 3:**
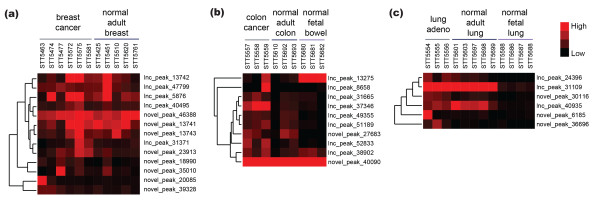
**Significant lncRNAs and novel transcripts in breast, lung and colon cancer**. LncRNAs and novel transcripts significantly differentially expressed in **(a) **breast (*n *= 13), **(b) **colon (*n *= 10) and **(c) **lung (*n *= 6) cancers. Normalized, uncentered read data for cancer and normal samples were hierarchically clustered and plotted on low (black) to high (red) heatmaps.

Although most of the peaks showed some level of expression in the normal samples (929 lncRNAs (87%) and 867 novel peaks (81%) showed greater than 0.4 expression in at least one of the normal samples), generally the maximally expressing cancer sample showed higher expression levels than the maximally expressing normal sample (888 lncRNAs (83%) and 955 novel peaks (89%)). However, average expression levels for the transcripts across the cancer and normal samples tended to be comparable (R = 0.9635 for lncRNAs and R = 0.9329 for novel peaks; see Figure S3 in Additional file 2). Because this analysis did not include normal tissue-of-origin matches for several of the cancer types, we were interested in further evaluating expression levels for transcripts in the subset of cancers with corresponding normal tissues (breast, colon and lung). Specifically, were the transcripts identified as highly expressed and specific for cancer subtypes expressed in their normal tissue counterparts? Indeed, for those peaks differentially expressed in cancer (13 in breast, 10 in colon, 6 in lung), although the average expression of the peaks tended to be higher in cancer tissues, most of the peaks showed evidence of expression in normal tissues (Figure 3). Interestingly, the breast cancer peaks showed more comparable expression levels between cancer and normal tissue, whereas the colon cancer peaks were expressed at lower levels in normal adult tissue. Two of the colon cancer peaks showed distinct normal fetal expression, whereas none of the lung-specific peaks showed obvious fetal expression. Two of the lung cancer peaks were highly expressed in normal adult lung tissue. This case study of a small number of normal counterparts showed that, although expression patterns between cancer and normal tissue may vary, the cancer peaks identified in this study tended to show some level of normal expression. These findings suggest that many of these transcripts may have functional roles in normal tissues and their expression is maintained in the corresponding cancers.

### A case study of breast-specific RNAs

**Figure 4:**
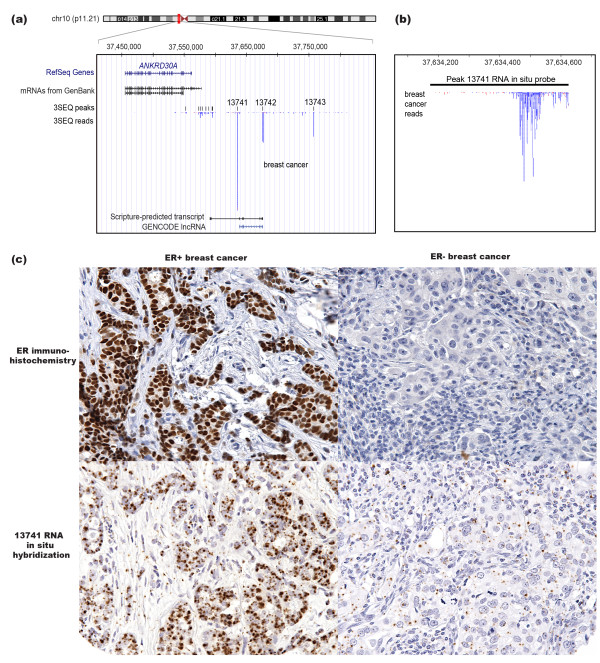
**A case study of novel, breast-specific peak 13741**. **(a) **Browser shot showing expression for a breast cancer sample in the region downstream of *ANKRD30A *on chromosome 10. The first two tracks show the known genes and RNAs in this locus. The third track shows the peaks identified in this study, including three highly expressed peaks: novel 13741, lncRNA 13742 and novel 13743. The fourth track shows the raw 3SEQ reads (transcript abundance levels) on the forward strand (blue) and reverse strand (red). The final tracks show the longest transcripts that overlap peak 13742, a Scripture-assembled transcript produced using normal breast RNAseq reads from the Illumina BodyMap data set and GENCODE lncRNA ENSG00000235687. **(b) **Zoom-in browser shot of peak 13741 on chromosome 10 shows the location of the RNA *in situ *hybridization probe (top track) as well as the raw sequence reads for one breast cancer sample (bottom track). This peak illustrates the shape of a typical 3SEQ peak from a high-expressing transcript. **(c) **ER staining on an ER+ breast cancer (top left) and an ER-breast cancer (top right). RNA *in situ *hybridization for peak 13741 performed on the same ER+ breast cancer specimen (bottom left) and same ER- breast cancer (bottom right). Specimens were matched but ER and 13741 stains used different tissue slices. All images are at 400× magnification. 3SEQ, 3'-end sequencing for expression quantification; chr10, chromosome 10; ER, estrogen receptor; lncRNA, long non-coding RNA.

We wished to confirm and extend our observations about lncRNA expression patterns obtained using 3SEQ by exploring the expression of one of our novel transcripts using an alternative expression method, RNA *in situ *hybridization. Our 3SEQ profiling efforts relied on RNA collected from the various cell types comprising heterogeneous tissue and tumor samples. We hypothesized that lncRNA expression is cell-type specific within normal tissues and that this spatial specificity will be recapitulated in the cancer counterparts. RNA *in situ *hybridization performed on tissue microarrays allows a quick method for assessing expression of an RNA of interest across numerous samples in parallel as well as providing additional information about how the RNA is expressed across cell types and how it is localized within cells. To this end, we identified an intriguing set of three breast-specific transcripts (two novel and one lncRNA) showing particularly high expression levels and located within a 200 kb intergenic region on chromosome 10, approximately 50 kb downstream of the nearest protein-coding gene, the breast gene *ANKRD30A *(also known as *NY-BR-1*; Figure 4a[25]). An analysis of 3SEQ data from these three peaks, 13741, 13742 and 13743, showed that they were variably expressed in both normal breast and breast cancer tissue, but were not expressed in cancers from other tissue types (Figure S4a in Additional file 2). Because peak 13741 was the most highly expressed of the three 3SEQ peaks in the region, we further studied this RNA. Peak 13741 expression was examined in a panel of 11 breast cancer cell lines using qRT-PCR (Figure S4b in Additional file 2), and northern blot analysis was used to determine that peak 13741 corresponded with a long transcript, greater than 5 kb (Figure S4c in Additional file 2).

The genomic coordinates of the 13741 transcript were unclear because its 3SEQ peak did not overlap any annotated exons in known genes or pseudogenes. Peak 13742 was annotated as a known lncRNA in our analysis because of its overlap with GENCODE transcript ENSG00000235687 (Figure 4a). Peak 13742 also corresponded with a spliced transcript predicted by Scripture using the BodyMap normal breast dataset. To further study the transcripts within this locus, we performed transcript prediction using RNAseq data we generated from six breast cancer cell lines expressing peak 13741 and identified a number of predicted transcripts in this region (Figure S5 in Additional file 2; [GEO:GSE28866]). Interestingly, most of the spliced transcripts corresponded with the 13742 transcript, leaving us with only short single-exon transcript predictions (some of them within introns of the 13742 transcript isoforms) to explain peaks 13741 and 13743. Additionally, the genomic region containing 13741 did not appear to be evolutionarily conserved, nor did it span any repetitive elements.

Though the full transcript structure for peak 13741 remains to be determined, we used the genomic sequence spanning peak 13741 to develop an RNA *in situ *hybridization probe (Figure 4b) and evaluated the expression of peak 13741 on several cancer tissue microarrays. The RNA *in situ *hybridization signal representing peak 13741 expression for both invasive breast cancer and normal breast tissue was confined to the epithelial cells within the samples (Figure 4c, bottom left). Within the normal breast tissues examined, expression varied and was present in 5% to 30% of cells. Strong staining was observed in 127 out of the 197 invasive breast carcinomas, as defined by greater than 30% of cells staining, though 81.9% (104 out of 127) showed staining in the vast majority of the tumor cells (60% to 95% of tumor cells staining). The probe signal for all of the positively staining samples appeared localized to the nucleus (Figure 4c, bottom left), which is distinct from the cytoplasmic probe signal typically observed when RNA *in situ *hybridization is performed on protein-coding mRNAs. Instead, this nuclear pattern follows the trend for lncRNAs and other regulatory RNAs, which, with limited evidence, tend to show nuclear localization [26].

Although the structure and function of the transcript corresponding with peak 13741 remains to be determined, we were struck by this transcript's potential as a breast cancer biomarker because of the specificity of its expression across the more than 800 samples surveyed. Specifically, we found that significant expression was only present in breast tissue, with peak 13741 showing no significant expression in other neoplasms or normal tissues. Because a range of breast cancer subtypes were present on the tissue microarrays, we were able to determine that the expression of peak 13741 was positively associated with low tumor grade (*P *= 6.1 × 10^-8^), estrogen receptor (ER) expression (Figure 4c, top panels, *P *= 6.4 × 10^-15^) and progesterone receptor (PR) expression (*P *= 6.8 × 10^-14^) in breast cancers and showed no significant association with metastasis or human epidermal growth factor receptor 2 (HER2) expression (Table 1). That the expression was correlated with ER+ or PR+ breast cancers and was largely absent from ER- tumors suggests that the transcript corresponding to peak 13741 may function specifically within normal breast tissue and low grade ER+ or PR+ breast cancer. Thus, it may be useful not only as a biomarker to distinguish breast cancer subtypes but also as an interesting, possibly regulatory RNA in normal breast and breast cancer biology.

**Table 1:** Novel, breast-specific peak 13741 is associated with estrogen-receptor-positive and progesterone-receptor-positive cells and Grade 1 breast cancer

	ER+	ER-	PR+	PR-	Grade 1	Grade 3
	(*n *= 146)	(*n *= 33)	(*n *= 129)	(*n *= 50)	(*n *= 42)	(*n *= 41)
13741+	122 (83.6%)	5 (15.2%)	112 (86.8%)	15 (30.0%)	37 (88.1%)	12 (29.3%)
						
13741-	24 (16.4%)	28 (84.8%)	17 (13.2%)	35 (70.0%)	5 (11.9%)	29 (70.7%)
						

RNA *in situ *hybridization results for analyses correlating the expression of peak 13741. Numbers of samples are reported. Each test was significant by the Kruskal-Wallis rank sum test. +, staining in >30% of cells; -, staining in <10% of cells; ER, estrogen receptor; PR, progesterone receptor.

## Discussion

Recent studies have demonstrated the importance of lncRNAs in regulating embryogenesis and gene expression, and there is considerable evidence that this class of RNAs plays important roles in cancer [14,18,19,27,28]. However, the definition of lncRNAs is still evolving and even the number of lncRNAs encoded by the human genome is unclear, making it difficult to survey lncRNA expression comprehensively. We surveyed lncRNA expression in cancer by using 3SEQ, a 3'-end targeted RNAseq method, to determine how lncRNAs are expressed within cancers and their normal counterparts, and to identify novel intergenic transcripts in cancers.

Several groups have compiled catalogs of lncRNAs, ranging from lists of a few hundred named lncRNAs to lists of up to 15,000 predicted lncRNAs [12,13]. We compared our peaks with four of the larger lncRNA catalogs. Three of these catalogs themselves include compilations of previous catalogs and datasets. Ulitsky and colleagues assembled a set of 2,458 lncRNAs by computationally filtering transcripts from the RefSeq, Ensembl and UCSC gene lists [10]. Cabili and colleagues assembled a catalog using 3,376 lncRNAs from RefSeq, GENCODE and UCSC and adding 4,819 lncRNAs identified from among the predicted transcripts assembled from the Human Body Map RNAseq dataset [4]. Versions 7 to 10 of the GENCODE lncRNA annotation set, assembled using manual curation, computational analysis and targeted experimental validation of GENCODE transcripts compiled by the ENCODE project, comprise more than 15,000 transcripts representing more than 9,000 non-overlapping lncRNA sequences (Derrien *et al*., submitted) [5,13,29]. van Bakel and colleagues used a normal human RNAseq dataset to predict approximately 1,250 novel intergenic lncRNA transcripts [11]. Because the various catalogs were based on different datasets and employed different criteria for lncRNA prediction, perhaps it is not surprising that they did not show high percentages of transcript overlap. In addition, these studies were based on transcripts primarily isolated from normal tissues and cell lines; no study to date has evaluated these newest annotated lncRNAs for expression in human cancers. In this study, we used the union of all of the lncRNAs from the four catalogs described above as the reference set for comparison and showed that 1,065 of the 3SEQ peaks expressed in our cancer panel overlap previously predicted lncRNAs. This allowed us to determine for the first time the expression patterns for many of these lncRNAs within primary tumor samples.

We also found 1,071 novel intergenic 3SEQ peaks expressed in our cancer panel. These RNAs were isolated using the same polyA+ and size selection used to capture the lncRNAs and the >21,000 protein-coding mRNAs. We therefore inferred that the novel intergenic peaks were derived from long (>200 bp) polyA+ transcripts; however, overlap analysis with Scripture-predicted transcripts suggested that some of these novel transcripts were single-exon transcripts. Although both the levels and patterns of gene expression across the novel intergenic peaks appeared similar to the lncRNA peaks, suggesting that these novel peaks may be new lncRNAs, these peaks also resembled the short intergenic transcripts described by others [11]. Further characterization of these novel transcripts will be required to determine their structure and functional importance within cancer.

Profiling of the non-coding transcriptome in primary human cancers has been limited, despite the evidence that lncRNAs are important players in gene regulation. We therefore performed unbiased expression profiling on a diverse panel of solid cancers, representing 17 diagnostic subclasses of adenocarcinomas, squamous cell carcinomas and sarcomas. We identified 267 lncRNAs and 217 novel transcribed regions that were differentially expressed in at least one of the cancer types. Differential expression has been used as an important filtering parameter for identifying genes that are not only correlated with but potentially functionally important in various cancer types. Despite the limited number of samples representing each diagnosis, we were struck by the extent of specificity of some of these transcripts. Though many of the transcripts appeared to show some expression in the normal samples examined, these differentially expressed transcripts are intriguing candidates for both future study and more immediately for biomarker analyses.

Because previous cancer studies performed on lncRNAs have been small-scale studies using a less comprehensive collection of cancer samples, it has been difficult to make any generalizations about whether the expression of lncRNAs differs between cancer and normal tissue. We took the opportunity to address this question by analyzing 3SEQ expression for the cancer-expressed lncRNA peaks and novel transcripts within 27 normal tissues. Although our analysis was limited to only a handful of types of normal tissues, we observed that the lncRNAs profiled in this study and expressed in cancer tended to have at least some expression in the normal samples. Therefore, the cancer-expressed lncRNAs may not to be specific to cancer, but most likely have some function in normal tissues. It also does not appear that lncRNAs, as a class, have significantly different expression levels between cancer and normal tissue. More striking, however, was that many cancer-expressed lncRNAs and novel transcripts showed tissue-specific expression patterns and, for some cancers, the normal tissue-of-origin expression patterns appeared to be maintained in the associated cancers. Further research is needed to determine the function and importance of the tissue-specific lncRNAs in both cancer and normal biology.

RNA *in situ *hybridization allowed us to further characterize the expression of a novel breast-specific transcript, peak 13741, across hundreds of cancer and normal patient samples. We demonstrated that the transcript was not only tissue-specific (expressed only in breast and breast cancers), it was cell-type specific (expressed in epithelial breast cells and not the surrounding stromal cells) and had specific cellular localization patterns (nuclear, as opposed to cytoplasmic). All of these observations suggest that this transcript has regulated transcription and may have a functional role within breast tissue biology. The correlated expression of this transcript with ER in breast cancer and the apparent loss of expression of this transcript in ER- breast cancer suggest that this transcript may be functioning within the ER pathway. Further research is necessary to determine the function of the peak 13741 transcript, because of its strong correlation with ER+ and PR+ breast cancers, but this peak has potential as a useful breast cancer biomarker.

## Conclusions

This study represents the first large survey of lncRNA expression across a diverse panel of primary human cancer samples and provides the research community with a valuable resource for cancer-expressed lncRNAs as well as a collection of novel transcripts expressed within cancers. We illustrate that 3SEQ can be used for transcriptional profiling to identify both known lncRNAs and novel transcripts within archived primary tissue samples, and our RNA *in situ *hybridization analysis of one such novel RNA, peak 13741, identified it as an intriguing breast-specific RNA that may be important for the differences between ER+ and ER- breast cancers.

## Methods

### Tissue samples

Tumor and normal samples were collected using written informed consent compliant with the Health Insurance Portability and Accountability Act and approved by the Stanford University Medical Center institutional review board. Some of the tissues already existed in tissue banks and fell under exemption 4. For this study, we selected 64 formalin-fixed paraffin-embedded (FFPE) cancer samples, along with 27 normal samples, following examination of hematoxylin and eosin stains by pathologists RBW and MvdR. Multiple 2 mm-diameter cores were taken from each FFPE block, re-embedded in paraffin at a perpendicular orientation, and then sliced into 4-μm-sections for further hematoxylin and eosin examination or 20-μm-sections for nucleic acid extraction. Approximately one hundred 20-μm-slices were placed into one to two 1.5 mL tubes for nucleic acid isolation. (See Table S1 in Additional file 1 for sample numbers and diagnoses.)

### RNA isolation and 3SEQ library preparation

RNA was purified from FFPE slices following deparaffination with a xylene incubation, ethanol wash and protease digestion, using the RecoverAll Total Nucleic Acid Isolation Kit (Ambion/Life Technologies, Austin, TX, USA), as described previously [21]. Isolation of the 3'-ends of mRNA was achieved with an oligodT selection performed on at least 5 μg total RNA using the Oligotex mRNA mini Kit (QIAgen, Valencia, CA, USA). RNA that was not sufficiently fragmented was heat-sheared to a size of approximately 100 to 200 bp. The polyA+ RNA was then subjected to first and second strand cDNA synthesis and Illumina library synthesis, as described previously [21]. (Duplicate 3SEQ libraries were created for two cancer samples: ESSSTT5520 and LMS STT516.)

### Sequencing and mapping

3SEQ libraries were sequenced with Illumina Genome Analyzer IIx machines to obtain a minimum of 1.5 million uniquely mapping 25-base single-end sequence reads. Reads were mapped to hg18 with a two-mismatch allowance using ELAND (Illumina Inc., San Diego, CA, USA). Sequence reads were further filtered to remove mapping artifacts caused by ambiguous mapping by requiring a posterior probability of at least 0.8 for the best alignment, where posterior probability was calculated as the ratio of the likelihood of the best alignment to the sum of the likelihoods of all alignments. Likelihood was taken from a binomial model of sequencing errors with constant substitution rate 0.01. FASTQ files and filtered bed files of hg18-mapped reads are available in [GEO:GSE28866].

### Peak analysis

Detection of genomic regions enriched for sequencing reads (peak-calling) was performed once, by combining filtered sequence reads from all 66 cancer libraries and using a custom Perl script to call genomic regions with greater than 150 reads across all samples. This provided a single set of genomic peak coordinates, which allowed for direct comparison of all samples. Because of our interest in identifying peaks expressed in only a fraction of samples, we initially selected a more permissive overall threshold, corresponding with roughly a two-fold enrichment above that expected by chance when applying a uniform distribution model for reads from all samples across the mappable genome. During this process, clusters of sequence reads, each within 20 bp of their nearest same-strand neighbor, were identified and the coordinates for these genomic regions were defined using the start coordinates for the left-most and right-most sequence reads within each cluster. The list of peaks was then filtered to exclude any region that contained fewer than 25 different starting positions for the reads within the region, and in this way required each peak to possess a minimum of 25 unique reads. The initial list of 54,511 peaks was further filtered to remove peaks antisense to peaks in known genes (determined to be artifacts of library preparation). Applying a more stringent threshold requiring peaks to possess at least 200 total reads resulted in a filtered list of 36,048 peaks. Assuming a uniform distribution of reads to calculate expected background levels, we determined that peaks on this list were at least 2.5-fold enriched above background when all samples were considered or at least four-fold enriched above background in at least one sample. Most peaks were enriched well above this level (median = 74-fold enrichment; see Tables S3 and S4 in Additional file 1 for fold-enrichment values). Peaks were annotated as coding genes if they overlapped any exons within annotated coding genes from the RefSeq and UCSC known gene datasets. Peaks were then overlapped with genomic coordinates for the lncRNA transcripts from the following datasets: Ulitsky *et al. *(*n *= 2,642) [10]; Cabili *et al. *(*n *= 14,309) [4]; GENCODE lncRNA annotation sets version 7 (*n *= 15,207), version 9 (*n *= 18,878) and version 10 (*n *= 17,547) [5,13,29]; and van Bakel *et al. *(*n *= 1,252) [11]. The remaining peaks were determined to be novel intergenic peaks if they did not overlap promoter regions (within 5 kb), exons, introns or downstream regions (within 5 kb) of any known genes from the RefSeq, UCSC Known Genes, GENCODE or all_mRNA (UCSC genome browser) datasets. Overlap analysis was used to further characterize the novel transcripts using several datasets downloaded from the UCSC genome browser, including all_ests, spliced_ests, phastConsElements17way (17way conservation), phastConsElements28wayPlacMammal (28way conservation), rmsk (RepeaterMasker), wgRna (small nucleolar RNAs, miRNAs), GENCODE genes and rnaGene. Pseudogenes were obtained from Pseudogene.org [30,31]. Files with raw counts of the sequence reads within each 3SEQ peak, as well as the normalized peak data, are available in [GEO:GSE28866].

### Gene expression analysis

The number of sequence reads located within each peak was determined for each sample. This raw read count data were then normalized for each 3SEQ peak using the sequencing depth of each sample by scaling by the mean value of each sample. Data were further compressed to reduce outliers by taking the square root of each value. Correlations between technical replicate libraries were determined to be r^2 ^= 0.91 and 0.95. Differential expression was determined using a series of two-class SAM analyses [24]. For each of the 17 cancer types, a two-class comparison of the specific cancer type versus all other cancers was performed to determine differentially expressed genes at an FDR <0.05. Using a two-class FDR of 0.05 to define differential expression, we calculated an overall FDR for identifying a peak as differentially expressed in at least one of the 17 two-class SAM analyses (overall FDR = 0.04). LncRNA and novel transcript expression were hierarchically clustered using the average linkage method in Cluster3 and visualized using Java TreeView (Figures 2 and 3).

### Quantitative RT-PCR

cDNA was made from the same total RNA used for the 3SEQ libraries in this study using the DyNAmo™ cDNA Synthesis Kit (Finnzymes/ThermoScientific, Lafayette, CO, USA). Primers were designed against 23 peak sequences using Primer3 and qRT-PCR was performed using the SYBR green method on a StepOnePlus instrument (Applied Biosystems/Life Technologies, Foster City, CA, USA) according to the manufacturer's instructions. Data were normalized using a series of five housekeeping genes (*ARL8B, CTBP1, CUL1*, *PAPOLA *and *ACTB*). For qRT-PCR on breast cancer cell lines, cDNA was amplified using primers against 13741 as described above and normalized using *ACTB*. See Table S6 in Additional file 1 for primer sequences.

### Breast cancer cell lines

HCC1419, HCC1500, UACC-812, ZR-75-30, CAMA-1, MCF7, MDA-MB-231, MDA-MB-361, MDA-MB-436 and MDA-MB-468 were obtained from American Type Culture Collection (Manassas, VA, USA), whereas SUM 44PE and SUM 52PE were a kind gift from Dr Stephen Ethier. Cells were grown to 70% to 80% confluence, and total RNA was isolated using the QIAgen RNeasy Mini Kit (QIAgen).

### RNAseq on breast cancer cell lines

Six breast cancer cell lines were used for transcriptome sequencing. RNAseq libraries were prepared using the Illumina mRNA-Seq Sample Prep Kit (Illumina, Inc.) as described previously [32]. Illumina Genome Analyzer IIx machines were then used to sequence paired-end 36mer reads, which were subsequently mapped to hg18 using TopHat and assembled into predicted transcripts using Scripture [6]. RNAseq data for the breast cancer cell lines are available in [GEO:GSE28866].

### Northern blot

Five micrograms of total RNA from UACC-812 and MDA-MB-436 were used as directed in the NorthernMax Kit (Life Technologies, Grand Island, NY, USA). To generate radioactive RNA probes, primers with SP6 polymerase binding sites were designed to amplify genomic DNA at the loci for 13741 and β-actin (*ACTB*) in a strand-specific manner (see Table S7 in Additional file 1 for primer sequences). *In vitro *transcription reactions were carried out using [ -^32^P]-UTP as directed in the SP6 MAXIscript Kit (Life Technologies). Following 13741 probe hybridization, the blot was stripped and re-probed for β-actin. The northern blot was exposed to maximum sensitivity film at -80°C with an intensifier screen for 24 hours for the 13741 hybridization and maximum resolution film for 2 hours for the β-actin hybridization.

### *RNA *in situ *hybridization*

The RNA *in situ *hybridization probe for peak 13741 was designed against chr10:37,634,173-37,634,625 (hg18) using primer 5'-TTGGAAAGCCAAATTGTTGA-3' and the T7 promoter-tagged primer 5'-CTAATACGACTCACTATAGGGTGTTTGTGTTCCCCCATTTT-3'. The digoxigenin-labeled antisense RNA probe was generated by PCR amplification and *in vitro *transcription using the DIG RNA labeling kit and T7 polymerase according to the manufacturer's protocol (Roche Diagnostics, Indianapolis, IN, USA). RNA *in situ *hybridization using the 13741 probe on a tissue microarray containing 197 breast cancers with clinical outcome data was performed as described previously [33]. Controls included known negative tissue types and other probes that were non-reactive. Additional tissue microarrays also tested for 13741 staining included ductal carcinoma *in situ *(*n *= 253), normal breast (*n *= 30) and a collection of other normal specimens (*n *= 17) including skin, adrenal gland, kidney, placenta, gallbladder, ovary, prostate, pancreas, salivary gland and thymus. Neoplasms tested for 13741 staining (*n *= 402) were from esophagus, liver, stomach, pancreas, kidney, colon, ovary, bladder, testes, skin, prostate, lung and uterus. Positive signal was defined as staining in at least 30% of tumor cells; staining in less than 10% of tumor cells within a case was considered negative. Several metrics were tested for association with expression of peak 13741, including ER expression, PR expression, tumor grade, human epidermal growth factor receptor 2 expression and the presence of metastasis using the Kruskal-Wallis rank sum test. Only Grade 1 and 3 tumors were used in the association with peak 13741.

## Abbreviations

3SEQ: 3'-end sequencing for expression quantification; bp: base pairs; ER: estrogen receptor; EST: expressed sequence tag; FDR: false discovery rate; FFPE: formalin-fixed paraffin-embedded; lncRNA: long non-coding RNA; miRNA: microRNA; PR: progesterone receptor; qRT-PCR: quantitative reverse transcription polymerase chain reaction; RNAseq: RNA sequencing; SAM: significance analysis of microarrays; UCSC: University of California: Santa Cruz.

## Competing interests

The authors declare that they have no competing interests.

## Authors' contributions

ALB, AHB and RBW designed the study, analyzed the data, and wrote the paper. ALB, BE and TG conducted the qRT-PCR experiments. RTS scored the RNA *in situ *hybridization. SXZ, RL, KM and SV processed the tissue blocks, purified RNA and built the 3SEQ libraries. RL also designed and performed the *in situ *hybridization experiments. XG, JWF, DMW and RT assisted with data processing, normalization and analysis. JRP and CPG contributed RNA as well as RNAseq data from the breast cancer cell lines. RAF performed the northern blot. HYC and MvdR assisted with designing the study and writing the paper. All authors read and approved the final manuscript.

## Supplementary Material

Additional file 1 **Supplemental tables 1-7**. Table S1 shows the sequencing statistics for the 3SEQ libraries. Table S2 shows the numbers of 3SEQ peaks overlapping lncRNAs and other annotation classes. Table S3 lists the 1,065 known lncRNAs. Table S4 lists the 1,071 novel intergenic peaks. Table S5 shows the number of peaks differentially expressed in each diagnostic class. Table S6 includes primer sequences used in qRT-PCR experiments. Table S7 includes primer sequences used for the northern blot probes. Click here for file

Additional file 2 **Supplemental figures 1-5**. Figure S1 plots sequencing depth versus RefSeq transcripts detected by 3SEQ. Figure S2 shows differential expression for the 23 peaks examined by qRT-PCR. Figure S3 plots the mean expression in cancer versus the mean expression in normal samples. Figure S4 shows expression of peak 13741 by 3SEQ, qRT-PCR and northern blot. Figure S5 is a browser shot showing the predicted breast transcripts near peak 13741. Click here for file
