# Supplementary material for: Transcriptional profiling of long non-coding RNAs and novel transcribed regions across a diverse panel of archived human cancers
Source: Genome Biol. 2012 Aug 28;13(8):R75. doi: 10.1186/gb-2012-13-8-r75 (PMC4053743; doi:10.1186/gb-2012-13-8-r75)
Supplement: Additional file 2 — Supplemental figures 1-5. Figure S1 plots sequencing depth versus RefSeq transcripts detected by 3SEQ. Figure S2 shows differential expression for the 23 peaks examined by qRT-PCR. Figure S3 plots the mean expression in cancer versus the mean expression in normal samples. Figure S4 shows expression of peak 13741 by 3SEQ, qRT-PCR and northern blot. Figure S5 is a browser shot showing the predicted breast transcripts near peak 13741. [file gb-2012-13-8-r75-S2.PDF]

Figure S1. Sequencing depth versus detection of RefSeq transcripts

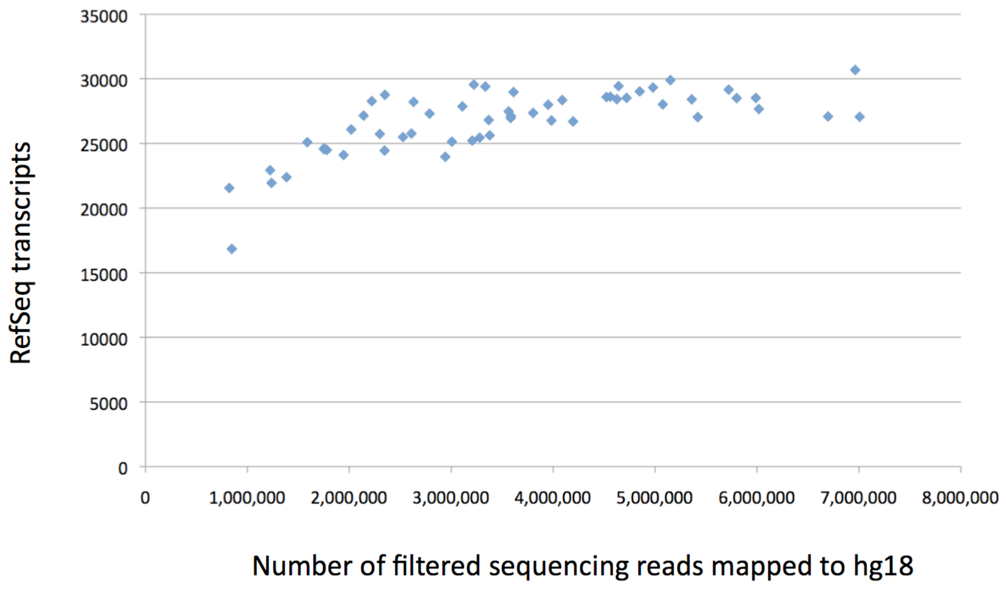

For each of the 66 libraries, all uniquely-mapping filtered reads were overlapped with the 37,576 RefSeq transcripts (hg18) downloaded from UCSC genome browser. Each library is plotted as a function of its read depth and the number of RefSeq transcripts overlapped by its sequence reads.

Figure S2. Differential expression of 23 peaks examined by qRT-PCR

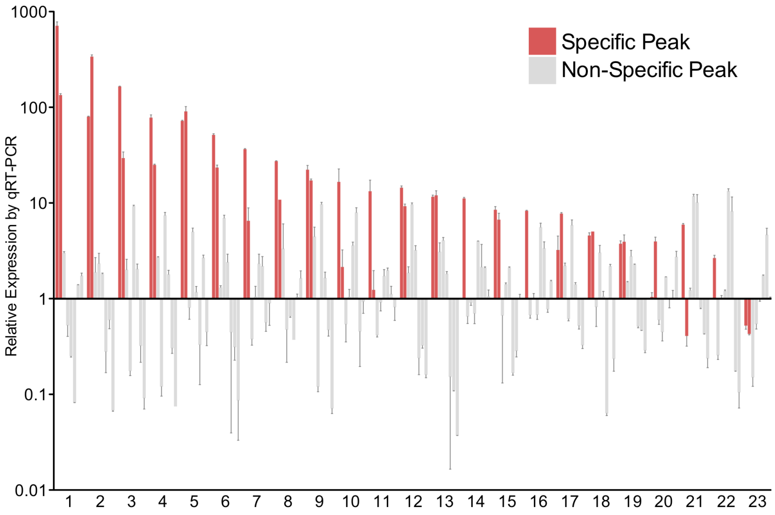

1.	Peak 24822	Prostate
2.	Peak 32284	EWS
3.	Peak 46776	SS
4.	Peak 46778	SS
5.	Peak 21093	GIST
6.	Peak 25147	EWS
7.	Peak 8522	NPC
8.	Peak 4657	Prostate
9.	Peak 46777	SS
10.	Peak 107	NPC
11.	Peak 15308	NPC
12.	Peak 46115	EWS
13.	Peak 781	EWS
14.	Peak 3175	PUC
15.	Peak 19508	Prostate
16.	Peak 30357	PUC
17.	Peak 18873	SS
18.	Peak 48219	GIST
19.	Peak 43785	ESS
20.	Peak 39524	HNSCC
21.	Peak 6380	ESS
22.	Peak 3787	PUC
23.	Peak 41198	SkinSCC

qRT-PCR was performed for each of the 23 peaks listed. The cancer subtype listed for each peak in the table showed increased expression relative to all other cancer subtypes in the 3SEQ data. The plot shows the qRT-PCR results when each of the 23 peaks was tested for expression in multiple cancer types. For each peak, the red bars indicate the tissue predicted to be more highly expressed by 3SEQ. The grey bars show other tissues. Data was normalized using the average expression of five housekeeping genes (*ARL8B, CTBP1, CUL1, PAPOLA,* and *ACTB*). The pair of red bars indicates when two independent samples were tested from the expected cancer subtype. Each PCR reaction was performed in duplicate; error bars indicate standard deviation. Reaction 1 (Peak 24822) corresponds to the known prostate gene *KLK4*. All other peaks correspond to candidate lncRNA or novel peaks identified in the study. Experiments 1-19 show increased expression in the expected cancer subtype relative to the average expression of the other tissues tested. See Additional file 1, Table S6 for primer sequences.

Figure S3. Mean cancer expression versus mean normal expression

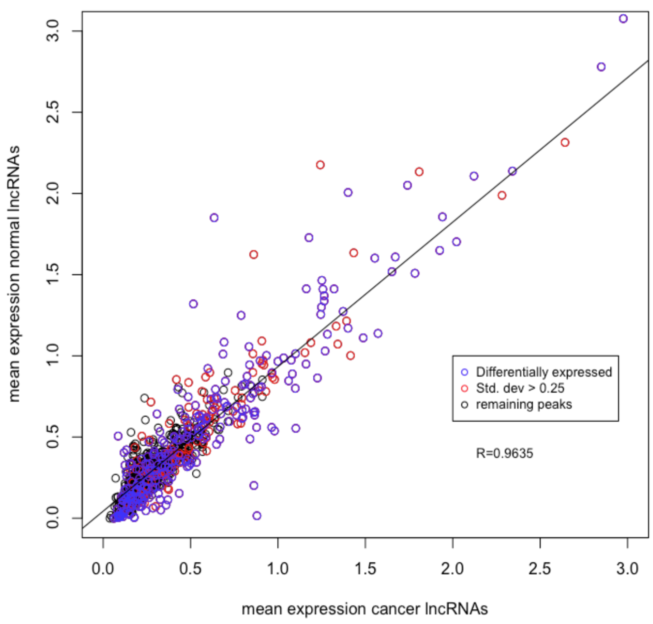

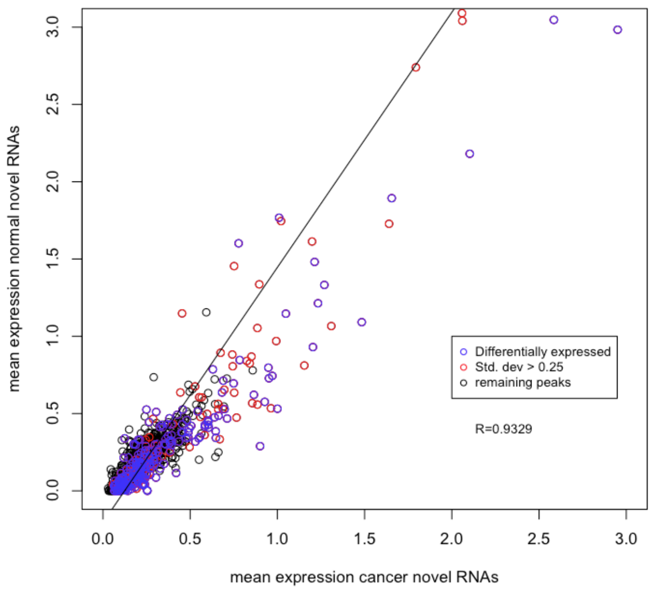

Mean expression across the 66 cancer samples versus mean expression across the 27 normal samples for lncRNAs (n=1,065, top) and novel peaks (n=1,071, bottom). Correlation coefficients are 0.9635 and 0.9329 for lncRNAs and novel peaks, respectively.

Figure S4. Expression of 13741 by 3SEQ, qRT-PCR, and Northern blot

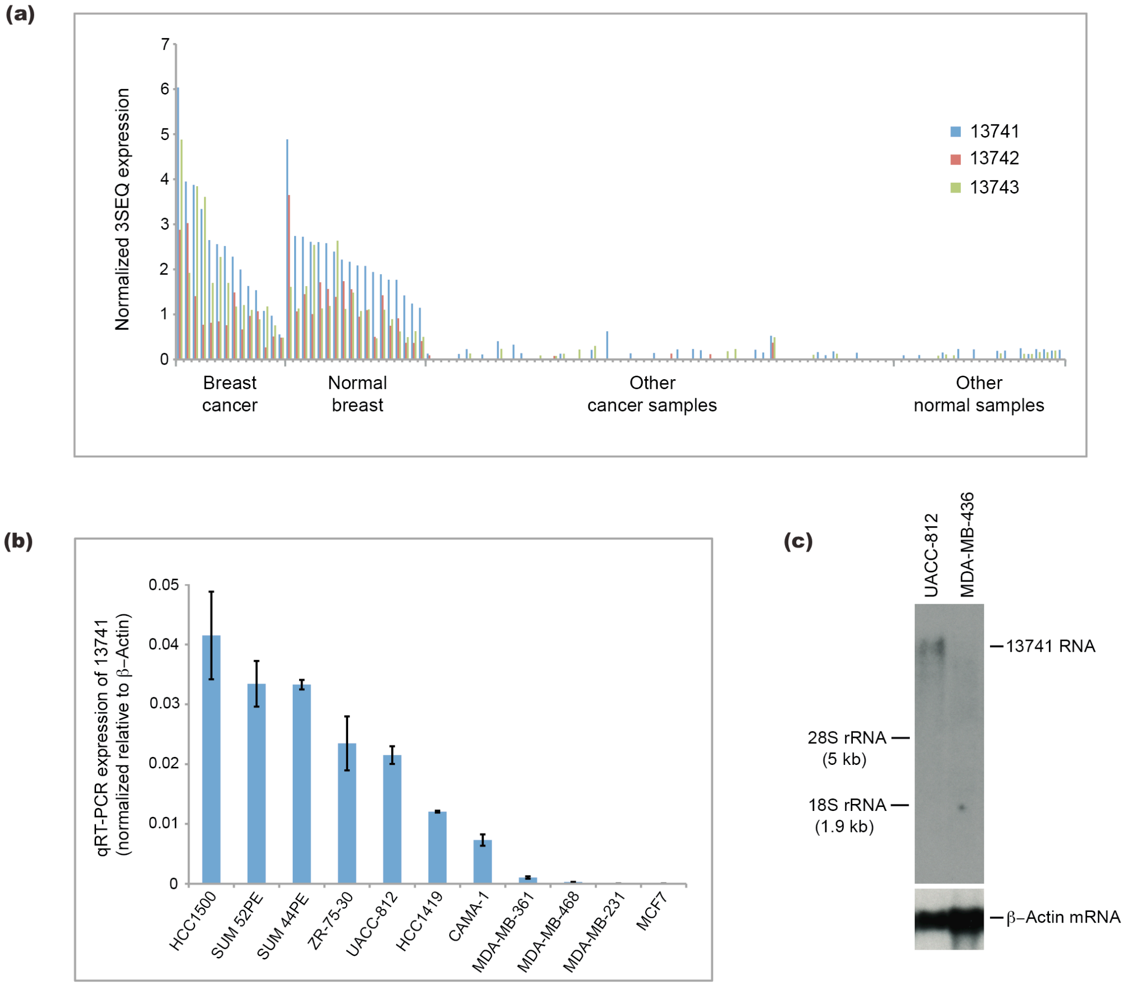

a) Normalized 3SEQ expression of Peaks 13741, 13742, and 13743 for 14 breast cancer samples, 18 normal breast samples, and the remaining cancer and normal samples. Non-breast samples are ordered as in Additional file 1, Table S1. Raw sequence count data was normalized by dividing each value by the sample mean, and then taking the square root. b) qRT-PCR expression for 13741 in 11 breast cancer cell lines. Values were normalized relative to *ACTB* expression. Error bars show the standard deviation of two replicate PCR reactions. See Additional file 1, Table S6 for primer sequences. c) Northern blot probed for 13741 expression (top) in two breast cancer cell lines. Max sensitivity film was exposed for 24 hours. The blot was stripped and re-probed for *ACTB* (bottom). Max resolution film was exposed for 2 hours. See Additional file 1, Table S7 for probe primer sequences.

Figure S5. Predicted breast transcripts near 13741

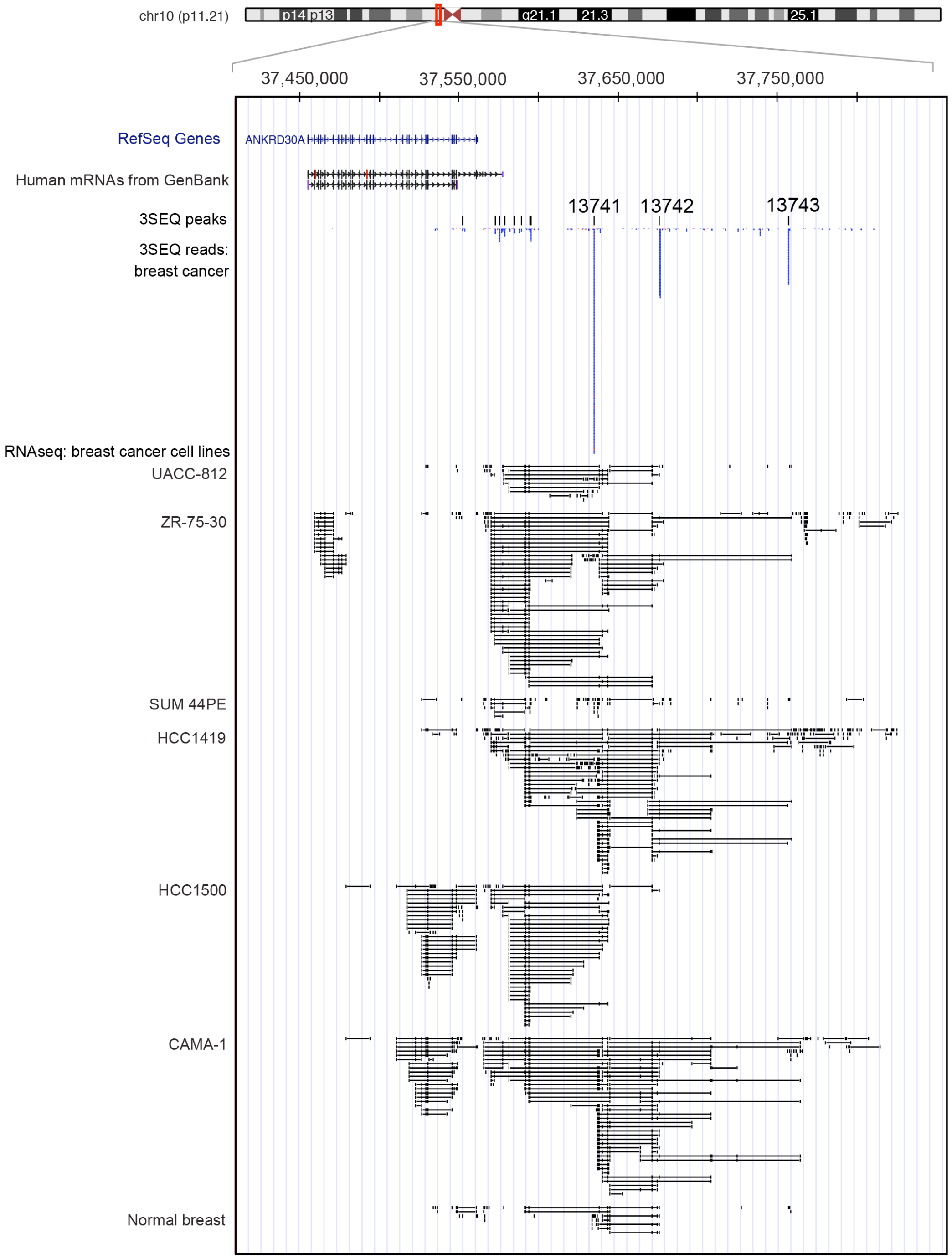

Scripture predictions using paired-end RNAseq from 6 breast cancer cell lines (UACC-812, ZR-75-30, SUM 44PE, HCC1419, HCC1500, and CAMA-1) and normal breast (Illumina BodyMap). Peak 13741 is located within an intron of the predicted spliced transcripts.
